# Multiple Signals Govern Utilization of a Polysaccharide in the Gut Bacterium *Bacteroides thetaiotaomicron*

**DOI:** 10.1128/mBio.01342-16

**Published:** 2016-10-11

**Authors:** Nathan D. Schwalm, Guy E. Townsend, Eduardo A. Groisman

**Affiliations:** a Department of Microbial Pathogenesis, Yale School of Medicine, New Haven, Connecticut, USA; b Yale Microbial Diversity Institute, West Haven, Connecticut, USA

## Abstract

The utilization of simple sugars is widespread across all domains of life. In contrast, the breakdown of complex carbohydrates is restricted to a subset of organisms. A regulatory paradigm for integration of complex polysaccharide breakdown with simple sugar utilization was established in the mammalian gut symbiont *Bacteroides thetaiotaomicron*, whereby sensing of monomeric fructose regulates catabolism of both fructose and polymeric fructans. We now report that a different regulatory paradigm governs utilization of monomeric arabinose and the arabinose polymer arabinan. We establish that (i) arabinan utilization genes are controlled by a transcriptional activator that responds to arabinan and by a transcriptional repressor that responds to arabinose, (ii) arabinose utilization genes are regulated directly by the arabinose-responding repressor but indirectly by the arabinan-responding activator, and (iii) activation of both arabinan and arabinose utilization genes requires a pleiotropic transcriptional regulator necessary for survival in the mammalian gut. Genomic analysis predicts that this paradigm is broadly applicable to the breakdown of other polysaccharides in both *B. thetaiotaomicron* and other gut *Bacteroides* spp. The uncovered mechanism enables regulation of polysaccharide utilization genes in response to both the polysaccharide and its breakdown products.

## IMPORTANCE

Breakdown of complex polysaccharides derived from “dietary fiber” is achieved by the mammalian gut microbiota. This breakdown creates a critical nutrient source for both the microbiota and its mammalian host. Because the availability of individual polysaccharides fluctuates with variations in the host diet, members of the microbiota strictly control expression of polysaccharide utilization genes. Our findings define a regulatory architecture that controls the breakdown of a polysaccharide by a gut bacterium in response to three distinct signals. This architecture integrates perception of a complex polysaccharide and its monomeric constituent as well as feedback of central metabolism. Moreover, it is broadly applicable to several prominent members of the mammalian gut microbiota. The identified regulatory strategy may contribute to the abundance of gut *Bacteroides*, despite fluctuations in the host diet.

## INTRODUCTION

A well-established paradigm governs the importation and metabolism of simple sugars in a wide range of bacterial species: expression of genes encoding transport and metabolic functions is induced by a specific sugar and prevented by other preferred sugars (1). For example, utilization of arabinose by the bacterium *Escherichia coli* requires a multisubunit transporter to import arabinose from the periplasm to the cytoplasm and enzymes to shunt arabinose into the pentose phosphate pathway (2). These proteins are encoded within four operons coordinately regulated by AraC, a protein that represses transcription of these operons in the absence of arabinose but activates their transcription when arabinose is present (3). Transcription of the arabinose utilization operons is independently stimulated by the cyclic AMP (cAMP) receptor protein (CRP) (3), a protein allosterically activated by cyclic AMP, the levels of which increase when preferred sugars are absent (1).

By contrast, the ability to import and metabolize complex polysaccharides exhibits a more limited phylogenetic distribution. Complex polysaccharide utilization is prominent in the *Bacteroidetes*, an abundant phylum within the mammalian gut microbiota (4–6). For example, in *Bacteroides thetaiotaomicron*, the fructose polymer levan is broken down to fructo-oligosaccharides that are transported into the periplasm and catabolized to monomeric fructose (7). A single regulatory protein activated by fructose promotes transcription both of genes necessary for the transport and degradation of levan and of genes necessary for transporting fructose into the cytoplasm and its entry into glycolysis (7).

**FIG 1:**
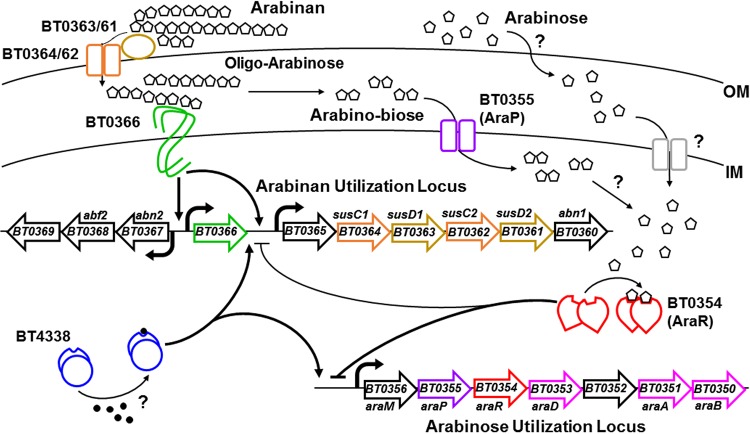
Schematic of the regulation of arabinan and arabinose utilization genes in *B. thetaiotaomicron*. Arabinan is extracellularly bound by two SusD-like proteins and imported into the periplasm by two SusC-like transporters. Large arabinan polymers are broken down into polymers of six to eight subunits in chain length (oligoarabinose) and eventually smaller arabino-oligosaccharides, such as arabinobiose, which are transported into the cytoplasm by AraP. These oligosaccharides are broken down to arabinose in the cytoplasm by an unknown glycoside hydrolase. Oligoarabinose binds to and activates the transcriptional regulator BT0366, which, in turn, promotes transcription of the arabinan utilization genes *BT0365* to -*60*, *BT0366*, and *BT0367* to -*69*. l-Arabinose is transported into the cell by an unknown mechanism. Cytoplasmic arabinose prevents binding of the transcriptional repressor AraR to the promoters of the arabinan utilization gene *BT0365* and the arabinose utilization gene *BT0356* (*araM*). The transcriptional regulator BT4338 is necessary for full activation of arabinan and arabinose utilization genes. The signal controlling the activity of BT4338 is at present unknown. OM, outer membrane; IM, inner membrane.

Analysis of the genes implicated in the utilization of the arabinose polymer arabinan and of monomeric arabinose suggests that their control is unlikely to follow the paradigm described for levan and fructose utilization because of the following. First, the sensing domain of the regulatory protein BT0366 binds arabinose polymers of six to eight subunits in length (4). Second, the *BT0366* gene is required for growth on arabinan (4, 8) but not arabinose (8) and for transcription of genes within the *BT0360*-to-*BT0369* arabinan polysaccharide utilization locus (PUL) (8). These genes encode proteins predicted to mediate transport and catabolism of arabinan (Fig. 1) (9–11). Third, putative arabinose utilization proteins are encoded within the independently transcribed *BT0356*-to-*BT0350* operon, which specifies proteins thought to transport arabinose into the cytoplasm and convert it to xylulose-5-phosphate, an intermediate in the pentose phosphate pathway (10, 11).

We now report the genetic basis for transcriptional control of arabinan and arabinose utilization genes. We determine that a regulatory protein activated by oligoarabinose in the periplasm controls transcription not only of arabinan utilization genes directly but also of arabinose utilization genes indirectly, by enabling the generation of arabinose, which allosterically inactivates a repressor of arabinose utilization genes in the cytoplasm. We establish that an inner membrane transporter encoded within the arabinose utilization locus is dispensable for growth on monomeric arabinose but is required for utilization of arabino-oligosaccharides. Furthermore, we uncover a role of a pleiotropic transcriptional regulator in the expression of both arabinan and arabinose utilization genes, and we demonstrate its requirement for the utilization of several carbohydrates. Taken together with the genome analysis of polysaccharide utilization in other *Bacteroides* species, our findings argue that the use of multiple regulators responding to different signals constitutes a new paradigm for the utilization of complex polysaccharides.

## RESULTS

### A permease encoded in the arabinose utilization operon is necessary for arabinan catabolism.

**FIG 2:**
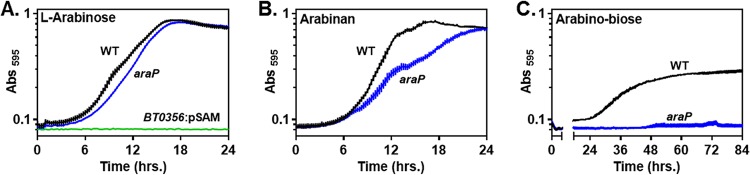
An *araP* mutant exhibits a significant growth defect on arabinan. (A) Growth of three *B. thetaiotaomicron* strains, one harboring a polar transposon insertion in the *BT0356* gene (*BT0356*:pSAM, NS423), one deleted for the *araP* gene (NS401), and the isogenic wild-type strain (WT, GT23), in minimal medium containing 0.5% arabinose. (B) Growth of isogenic *araP* strain (NS401) and wild-type *B. thetaiotaomicron* in minimal medium containing 0.5% arabinan. (C) Growth of isogenic *araP* strain (NS401) and wild-type *B. thetaiotaomicron* in minimal medium containing 0.5% arabinobiose. Graphed are the mean and standard error of the mean from at least five independent replicates grown in the same plate.

The *BT0356*-to-*BT0350* operon specifies functions necessary for l-arabinose utilization because a polar transposon insertion in the *BT0356* gene (Fig. 1) (12) prevented growth on l-arabinose (Fig. 2A) but not on fructose (see Fig. S1A in the supplemental material). Following *BT0356* is the *BT0355* gene, which encodes a putative permease designated AraP that is proposed to import arabinose from the periplasm into the cytoplasm (10, 11). Surprisingly, deletion of the *BT0355* (*araP*) gene resulted in a very modest decrease in growth rate on l-arabinose compared to the wild-type strain (average growth rate of 0.056 versus 0.061 Δ*A*_595_/h; *P* = 0.27 by Student’s two-tailed *t* test) (Fig. 2A). These results imply that a transporter other than AraP can import arabinose into the cytoplasm.

Because arabinose is generated during the breakdown of arabinose-containing polysaccharides, we hypothesized that *araP* is necessary for utilization of arabinan, a component of pectin comprised primarily of 1,5-linked arabinosyl residues (13), which is commonly found in the mammalian diet (14). As hypothesized, the *araP* mutant exhibited a growth defect on arabinan (Fig. 2B). The growth rate of the *araP* mutant was 0.035 Δ*A*_595_/h, significantly lower (*P* = 2.8 × 10^−5^ by Student’s two-tailed *t* test) than the wild-type growth rate of 0.067 Δ*A*_595_/h. However, the *araP* mutant reached the same final optical density as the isogenic wild-type strain by 24 h. By contrast, the *araP* mutant grew similarly to the wild-type strain in arabinogalactan (see Fig. S1B in the supplemental material), which contains terminal arabinofuranosyl side chains (13).

The results presented above suggested that AraP transports an intermediate in arabinan breakdown. In agreement with this notion, wild-type *B. thetaiotaomicron* grew on arabinobiose (the 1,5-linked α-l-arabinose disaccharide), albeit after a 24-h delay and to a lower growth yield than in l-arabinose or arabinan; however, the *araP* mutant did not grow on arabinobiose (Fig. 2C). Taken together, the results presented in this section establish that, despite being encoded in an arabinose utilization operon, AraP is necessary for normal arabinan catabolism and likely transports an arabino-oligosaccharide.

### Arabinan promotes transcription of both arabinan and arabinose utilization genes.

Because genes in the arabinose utilization locus are specifically involved in growth on arabinan (Fig. 2), we reasoned that the transcriptional activator of arabinan utilization genes, BT0366 (8), may control transcription of *araP* and other genes in the *BT0356*-to-*BT0350* operon. Thus, we examined the mRNA levels of both arabinan and arabinose utilization genes in isogenic wild-type and *BT0366* mutant strains following growth to mid-log phase in minimal medium containing 0.5% glucose as the sole carbon source and then switched to medium containing 0.1% arabinan.

**FIG 3:**
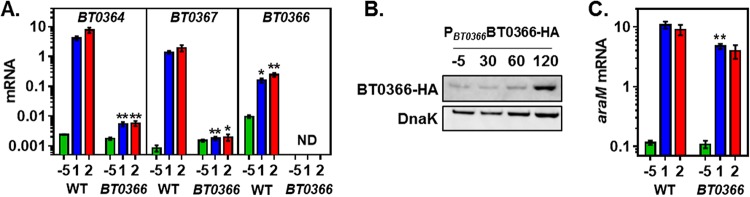
Arabinan promotes transcription of arabinan PUL genes and arabinose utilization genes in a *BT0366-*dependent manner. (A) mRNA levels of the *BT0364*, *BT0367*, and *BT0366* genes in isogenic *BT0366* (GT44) and wild-type (WT, GT23) *B. thetaiotaomicron* prior to the switch (−5) and after 1 and 2 h of exposure to minimal medium containing 0.1% arabinan. (B) Western blot of crude extracts from a strain specifying an HA-tagged BT0366 protein (NS204) collected from cultures grown to mid-log phase in minimal medium containing 0.5% glucose (−5) or 30, 60, and 120 min after switching to medium containing 0.1% arabinan. Data are representative of three independent experiments, which produced similar results. (C) mRNA levels of the *araM* gene in isogenic *BT0366* mutant (GT44) and wild-type (GT23) *B. thetaiotaomicron* prior to the switch (−5) and after 1 and 2 h of exposure to minimal medium containing 0.1% arabinan. Graphed are the mean and standard error of the mean from at least three independent experiments. Asterisks indicate significant differences from the wild-type strain for *BT0364* and *BT0367* expression and significant difference from the −5 sample for *BT0366* expression (*, *P* ≤ 0.05; **, *P* ≤ 0.01 by two-tailed Student’s *t* test). Note log scale of *y* axis in panels A and C.

The mRNA levels of the arabinan utilization genes *BT0364* and *BT0367* were >1,500-fold higher in the presence of arabinan than in the presence of glucose (Fig. 3A). Transcriptional activation of *BT0367* was absent in the *BT0366* mutant (Fig. 3A). By contrast, activation of *BT0364* was dramatically decreased, but not abolished, in the *BT0366* mutant (Fig. 3A). These results suggest that although *BT0364* and *BT0367* require *BT0366* for complete transcriptional activation by arabinan, they are differentially regulated.

The mRNA levels of the *BT0366* gene were 15-fold higher in arabinose than in glucose (Fig. 3A), which resulted in larger amounts of chromosomally encoded epitope-tagged BT0366 protein (Fig. 3B). These results suggested that the regulator of arabinan utilization genes positively regulates its own transcription.

The mRNA levels of the arabinose utilization gene *BT0356* (*araM*) increased 90-fold in the presence of arabinan (Fig. 3C). However, in contrast to the results obtained with the *BT0364* and *BT0367* genes (Fig. 3A), deletion of the *BT0366* gene decreased *araM* mRNA levels only 2-fold (Fig. 3C). The increase of *araM* mRNA levels observed in the *BT0366* mutant upon exposure to arabinan (Fig. 3C) appears to result from arabinose-containing polysaccharide contamination, because an ~30-fold increase in *araM* mRNA levels was still observed in this strain upon exposure to dialyzed arabinan (see Fig. S2A in the supplemental material). Moreover, the *BT0366* mutant retained wild-type growth on l-arabinose as the sole carbon source (see Fig. S2B). Cumulatively, these results, which are in agreement with previous reports (4, 8), establish that arabinan promotes transcription of arabinan and arabinose utilization genes and that this activation is strictly dependent on *BT0366* for the genes in the arabinan PUL but only moderately dependent on *BT0366* for the arabinose utilization genes.

### AraR represses transcription of arabinose utilization genes in the absence of arabinose.

Investigation of the *in vitro* properties of the regulatory protein AraR showed that it binds to DNA sequences located upstream of arabinose utilization gene *BT0356* and arabinan utilization gene *BT0365* (11) (see Fig. S3 in the supplemental material) and that binding to these DNAs was prevented when l-arabinose was present in the reaction mixture (11), suggesting that l-arabinose is an allosteric regulator of AraR. However, the *in vivo* function of AraR has remained unknown.

**FIG 4:**
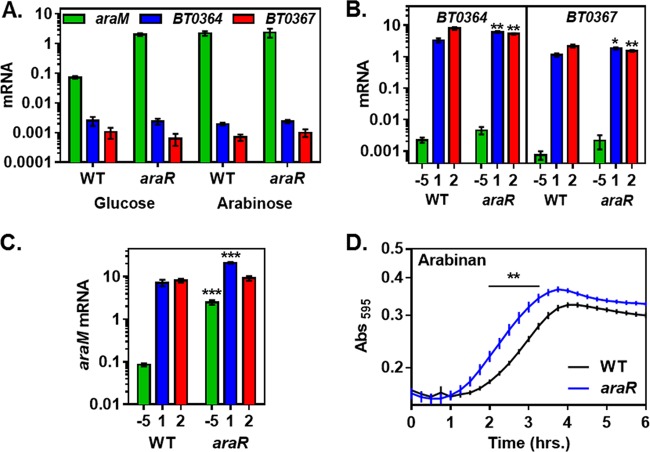
AraR is a repressor of arabinose and arabinan utilization genes. (A) mRNA levels of the *araM*, *BT0364*, and *BT0367* genes in isogenic *araR* (NS367) and wild-type (WT, GT23) *B. thetaiotaomicron* strains following growth in minimal medium containing 0.5% of either arabinose or glucose. (B) mRNA levels of the arabinan PUL genes *BT0364* and *BT0367* in isogenic *araR* (NS367) and wild-type (GT23) *B. thetaiotaomicron* strains prior to the switch (−5) and after 1 and 2 h of exposure to minimal medium containing 0.1% arabinan. (C) mRNA levels of the arabinose utilization gene *araM* in isogenic *araR* (NS367) and wild-type (GT23) *B. thetaiotaomicron* strains after 1 and 2 h of exposure to minimal medium containing 0.1% arabinan and prior to the switch (−5) to medium containing arabinan. (D) Growth of isogenic *araR* (NS367) and wild-type *B. thetaiotaomicron* (GT23) strains after switching from minimal medium containing 0.5% glucose to minimal medium containing 0.1% arabinan. Graphed are the mean and standard error of the mean from at least three independent experiments. Asterisks indicate significant difference from the wild-type strain (*, *P* ≤ 0.05; **, *P* ≤ 0.01; ***, *P* ≤ 0.001 by two-tailed Student’s *t* test). Note log scale of *y* axis in panels A, B, and C.

In wild-type *B. thetaiotaomicron*, *araM* mRNA levels were ~30-fold higher following growth in arabinose than in glucose (Fig. 4A). By contrast, an *araR*-deficient mutant displayed the same high mRNA levels during growth on arabinose and on glucose, which were similar to those observed in the wild-type strain grown in the presence of arabinose (Fig. 4A). Complementation of the *araR* mutant in *trans* restored *araM* transcription to the levels displayed by the wild-type strain (see Fig. S4A in the supplemental material). Taken together with the *in vitro* analysis of the AraR protein (11), our results indicate that AraR directly represses transcription of arabinose utilization genes and that this repression is antagonized by l-arabinose.

### AraR controls the transcription dynamics of arabinan utilization genes in the presence of arabinan.

Because arabinan catabolism generates arabinose, we wondered whether AraR might also regulate genes in the arabinan PUL, thereby providing feedback based on arabinose levels. However, the mRNA levels of the arabinan utilization genes *BT0364* and *BT0367* were not affected by inactivation of *araR* during growth on arabinose or glucose (Fig. 4A). Likewise, the *araR* mutant grew like the wild-type strain on arabinan (see Fig. S4B in the supplemental material).

The *araR* mutant exhibited altered expression dynamics of the *BT0364* and *BT0367* genes when *B. thetaiotaomicron* was switched from medium containing glucose to medium containing arabinan (Fig. 4B). In the wild-type strain, the mRNA levels of both *BT0364* and *BT0367* were >2-fold higher at 2 h than at 1 h after exposure to arabinan (Fig. 4B). By contrast, in the *araR* mutant, the mRNA levels of these two genes decreased between 1 and 2 h (Fig. 4B). These effects extend to genes necessary for the breakdown of arabinogalactan (see Fig. S4C in the supplemental material) but not rhamnogalacturonan I (see Fig. S4D), both of which contain arabinoyl residues. The mRNA levels of the arabinogalactan PUL gene *BT0268*, which encodes a SusC-like transporter similar to the *BT0364* product, were ~1.5-fold higher in the *araR* mutant than in the isogenic wild-type strain following 1-h exposure to arabinogalactan (see Fig. S4C). By contrast, mRNA levels of the rhamnogalacturonan I PUL SusC-like protein-encoding gene *BT4164* were nearly identical in the *araR* mutant and wild-type strains following exposure to rhamnogalacturonan I (see Fig. S4D). These arabinose-containing polysaccharides promoted an increase in the mRNA levels of the arabinose utilization gene *araM* (see Fig. S4C and D), presumably because arabinose is generated during their breakdown.

The mRNA levels of *araM* were only 3-fold higher in the *araR* mutant than in the wild-type strain 1 h after induction with arabinan, despite 30-fold-higher levels present during growth in glucose (Fig. 4C). However, the *araR* mutant displayed wild-type *araM* mRNA levels by 2 h (Fig. 4C). This is likely due to the breakdown of arabinan in the wild-type strain antagonizing AraR. The *araR* mutant initiated growth significantly faster than the isogenic wild-type strain following a switch from medium containing glucose to medium containing arabinan (Fig. 4D). This effect appears to be specific for arabinan because wild-type and *araR* strains grew similarly when switched to the unrelated polysaccharide chondroitin sulfate (see Fig. S4E in the supplemental material). Taken together, these results indicate that AraR is a transcriptional repressor that controls the expression kinetics of both arabinose and arabinan breakdown genes when *B. thetaiotaomicron* encounters arabinan.

### The arabinan-responsive BT0366 protein indirectly regulates arabinose utilization genes.

The BT0366 protein appears to control transcription of arabinose utilization genes in the presence of arabinan because the mRNA levels of the *araM* gene were lower in the *BT0366* mutant than in the isogenic wild-type strain (Fig. 3C). BT0366 may exert its regulatory effect directly, by binding to the *araM* promoter, or indirectly, by activating arabinan breakdown genes, thereby impacting cytoplasmic arabinose levels.

**FIG 5:**
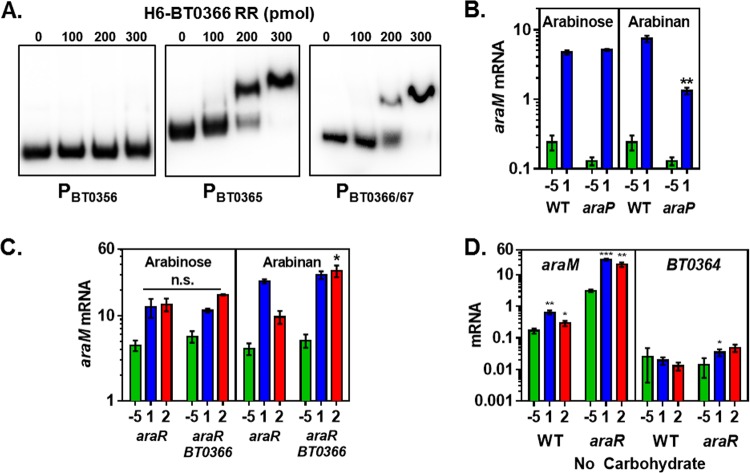
BT0366 controls expression of arabinose utilization genes but does not bind to the corresponding promoter regions. (A) Electrophoretic mobility shift assay (EMSA) of a fragment of the BT0366 hybrid two-component system harboring the response regulator and DNA-binding domains with DNA fragments located upstream of the *BT0365* coding region, intragenic to *BT0366-BT0367*, and upstream of the *araM* coding region. (B) mRNA levels of the *araM* gene in isogenic *araP* (NS401) and wild-type *B. thetaiotaomicron* (GT23) strains prior to the switch (−5) and after 1-h exposure to minimal medium containing 0.1% arabinose or 0.1% arabinan. (C) mRNA levels of the *araM* gene in isogenic *araR* (NS367) and *araR BT0366* (NS422) strains prior to the switch (−5) and after 1- and 2-h exposure to minimal medium containing 0.1% arabinan. (D) mRNA levels of the *araM* and *BT0364* genes in isogenic *araR* (NS367) and wild-type (GT23) strains after 1- and 2-h exposure to minimal medium lacking a carbohydrate. Graphed are the mean and standard error of the mean from at least three independent experiments. Asterisks indicate significant differences from the wild-type strain in panels B and C and significant difference from the −5 sample in panel D (*, *P* ≤ 0.05; **, *P* ≤ 0.01; ***, *P* ≤ 0.001 by two-tailed Student’s *t* test). Note log scale of *y* axis in panels B, C, and D.

BT0366 does not appear to control *araM* mRNA levels directly (i.e., by binding to the *BT0356*-to-*BT0350* promoter region), because the purified BT0366 protein did not shift a radiolabeled 207-bp fragment corresponding to the sequence immediately upstream of the *araM* start codon (see Fig. S3 in the supplemental material) in an electrophoretic mobility shift assay (EMSA) (Fig. 5A). By contrast, the BT0366 protein shifted the positive-control fragments (Fig. 5A) corresponding to the *BT0365* promoter and the *BT0367-BT0366* intragenic region (see Fig. S3).

In agreement with the notion that BT0366 controls *araM* mRNA levels indirectly (i.e., by generating the AraP substrate, which is broken down into arabinose), the *araP* mutant produced ~5-fold-lower *araM* mRNA levels than the wild-type strain when bacteria were switched from medium containing glucose to medium containing arabinan (Fig. 5B). The mRNA levels of *araM* were similar in the *araP* mutant and wild-type strains when switched to medium containing arabinose (Fig. 5B). As expected, the *araP* mutant retained wild-type *BT0364* mRNA levels upon induction with either arabinose or arabinan (see Fig. S5A in the supplemental material). These results suggest that BT0366 regulates transcription of arabinose utilization genes in the presence of arabinan by controlling the production of arabinose, which likely allosterically inactivates AraR.

### The absence of accessible carbohydrates promotes sustained transcription of the arabinose utilization gene *araM.*

We hypothesized that, when grown in arabinan, an *araR BT0366* double mutant would exhibit slightly lower *araM* mRNA levels than the *araR* single mutant. This is because the *araR* mutant exhibited higher *araM* mRNA levels than the wild-type strain in arabinan (Fig. 4C) and also because the *BT0366* mutant displayed 2-fold-lower *araM* mRNA levels under these conditions (Fig. 3C). To examine this possibility, we measured *araM* mRNA levels in isogenic *araR* and *araR BT0366* strains following exposure to arabinose or arabinan. The mRNA levels were similar in the two strains in arabinose (Fig. 5C), in agreement with the notion that *BT0366* is dispensable for arabinose utilization (see Fig. S3B in the supplemental material).

In arabinan, however, the *araR BT0366* double mutant exhibited sustained *araM* expression compared to the *araR* single mutant (Fig. 5C). That is to say, *araM* mRNA levels decreased between 1 and 2 h postinduction in the *araR* mutant but not in the *araR BT0366* double mutant. Because the *araR* single mutant grew on arabinan (see Fig. S4B in the supplemental material) but the *araR BT0366* double mutant did not (see Fig. S5B), the sustained *araM* expression exhibited by the latter strain may be triggered by lack of growth. In agreement with this notion, *araM* mRNA levels increased 9-fold in the *araR* mutant and 3-fold in the wild-type strain following a 1-h exposure to minimal medium lacking a carbohydrate (Fig. 5D). By contrast, *BT0364* mRNA levels remained essentially unchanged when bacteria were switched from medium containing glucose to medium lacking a carbohydrate (Fig. 5D). The latter results presumably reflect that in the absence of arabinan, the BT0366 protein does not promote *BT0364* transcription. Taken together, these results indicate that transcription of arabinose utilization genes responds to arabinose via AraR and to a signal produced under nutrient-poor conditions.

### The global regulator BT4338 controls *araM* transcription in the presence of arabinan.

In *E. coli*, transcriptional activation of arabinose utilization genes requires binding of both the AraC-arabinose complex and the CRP-cyclic AMP (cAMP) complex to target promoters (3). The N-terminal region of the *B. thetaiotaomicron* BT4338 gene product contains a CRP-like effector domain, and its C terminus harbors a helix-turn-helix DNA-binding motif (15). Moreover, a bioinformatics analysis predicted BT4338 binding to the *araM* promoter region (10). Therefore, we hypothesized that BT4338, originally named MalR for its role in maltose utilization in the absence of the starch utilization regulator SusR (16), operates as an activator of arabinose utilization genes.

**FIG 6:**
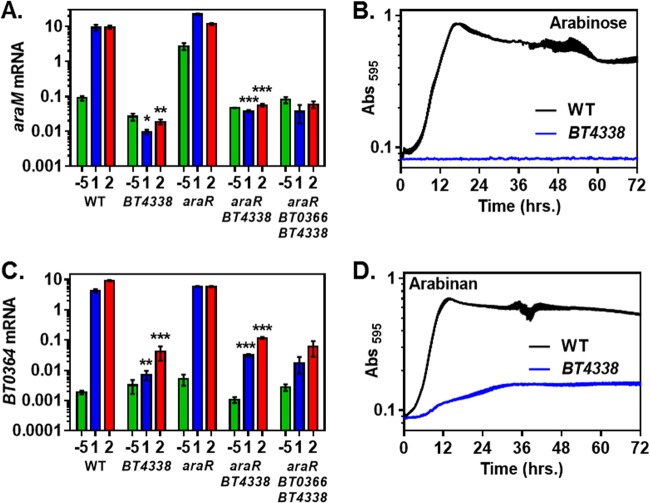
The regulatory gene *BT4338* is essential for arabinan utilization. (A) mRNA levels of the *araM* gene in isogenic wild-type (GT23), *BT4338* (NS364), *araR* (NS367), *araR BT4338* (NS404), and *araR BT0366 BT4338* (NS408) strains prior to the switch (−5) and after 1- and 2-h exposure to minimal medium containing 0.1% arabinan. (B) Growth of isogenic *BT4338* (NS364) and wild-type (GT23) strains in minimal medium containing 0.5% arabinose. (C) mRNA levels of the *BT0364* gene in isogenic wild-type (GT23), *BT4338* (NS364), *araR* (NS367), *araR BT4338* (NS404), and *araR BT0366 BT4338* (NS408) strains prior to the switch (−5) and after 1- and 2-h exposure to minimal medium containing 0.1% arabinan. (D) Growth of isogenic *BT4338* (NS364) and wild-type (GT23) strains in minimal medium containing 0.5% arabinan. For transcription experiments, the mean and standard error of the mean from at least three independent experiments are graphed. Asterisks indicate significant difference from the background strain containing *BT4338* (*, *P* ≤ 0.05; **, *P* ≤ 0.01; ***, *P* ≤ 0.001 by two-tailed Student’s *t* test). For growth experiments, graphed are the mean and standard error of the mean from at least five independent replicates grown in the same plate. Note log scale of *y* axis in panels A and C.

We examined *araM* mRNA levels in five isogenic strains—wild-type, *BT4338*, *araR*, *araR BT4338*, and *araR BT0366 BT4338* strains—following a switch from medium containing glucose to medium containing arabinan. Deletion of *BT4338* decreased the basal *araM* mRNA levels produced in glucose and abolished the induction promoted by arabinan (Fig. 6A). The *araR BT4338* double mutant and the *araR BT4338 BT0366* triple mutant displayed a similar behavior, though *araM* mRNA levels were 3- to 4-fold higher than in the *BT4338* single mutant (Fig. 6A).

The *BT4338* mutant was unable to grow on arabinose (Fig. 6B), reflecting its essential role in transcription of the arabinose utilization gene *araM* (Fig. 6A). By contrast, the *BT4338* mutant reached a wild-type growth yield in the rich tryptone-yeast extract-glucose (TYG) medium, albeit with slightly slower kinetics (see Fig. S6A in the supplemental material). Cumulatively, the results in this section establish that *BT4338* is required for transcriptional activation of arabinose utilization genes and that its role is not simply to overcome repression by AraR.

### The *BT4338* gene is necessary for full transcription of arabinan PUL genes.

Given the critical role that the *BT4338* gene plays in transcription of arabinose utilization genes, we investigated whether *BT4338* is also required for transcription of arabinan utilization genes. When *B. thetaiotaomicron* was exposed to arabinan, the mRNA levels of the arabinan PUL gene *BT0364* were ~600-fold higher at 1 h and ~225-fold higher at 2 h in the wild-type strain than in the *BT4338* mutant (Fig. 6C). The *BT4338 araR* double mutant displayed 3- to 4-fold-higher *BT0364* mRNA levels than the *BT4338* single mutant (Fig. 6C), analogous to the behavior of arabinose utilization genes (Fig. 6A). Furthermore, the *BT4338* null mutant was defective for growth on arabinan (Fig. 6D). Expression of the *BT4338* gene in *trans* from its native promoter and in single copy restored the ability of the *BT4338* mutant strain to grow in both arabinan (see Fig. S6B in the supplemental material) and arabinose (see Fig. S6C), albeit with slightly decreased kinetics. Taken together, these results demonstrate that *BT4338* is essential for *B. thetaiotaomicron* to utilize both monomeric arabinose and its polymeric form arabinan.

### The *BT4338* gene is required for growth utilizing a variety of carbohydrates.

Because the BT4338 protein has a domain structure similar to that of CRP, we explored the possibility of the *BT4338* gene being required for growth on carbohydrates other than arabinose and arabinan (Fig. 6B and D). The *BT4338* mutant displayed limited or no growth on arabinogalactan, fucose, glucuronate, *N*-acetylgalactosamine, polygalacturonic acid, ribose, or xylose (see Fig. S7A to G in the supplemental material). No difference in growth was observed between the *BT4338* mutant and the wild-type strain in glucose, heparin, mannose, or *N*-acetylglucosamine (see Fig. S7H to K). The *BT4338* mutant exhibited a longer lag phase than the isogenic wild-type strain in all other carbohydrates tested: amylopectin, chondroitin sulfate, fructose, galactose, galacturonate, maltose, maltotriose, α-mannan, pectic galactan, and rhamnogalacturonan I (see Fig. S7L to V). The difference in lag phase was short, ~2.6 h to an *A*_595_ of ≥0.2, in galactose (see Fig. S7L) but extended to ~24 h to an *A*_595_ of ≥0.2 in amylopectin (see Fig. S7M) and ~32 h to an *A*_595_ of ≥0.2 in galacturonate (see Fig. S7N). Taken together, these results indicate that *BT4338* both contributes to wild-type growth kinetics of *B. thetaiotaomicron* and is essential for growth on a variety of carbohydrates.

## DISCUSSION

We have uncovered how a gut bacterium integrates multiple signals to control expression of genes mediating the utilization of a polysaccharide and the monosaccharide derived from that polysaccharide (Fig. 1). The BT0366 hybrid two-component system of *B. thetaiotaomicron* senses an intermediate in arabinan breakdown in the periplasm and activates transcription of the arabinan PUL, which encodes products that transport arabinan into the cell and degrade it into arabinose.

Arabinose binding to the repressor AraR in the cytoplasm prevents AraR binding to the promoters of genes required for arabinose utilization and a subset of genes within the arabinan PUL (Fig. 4). The regulatory activities of BT0366 and AraR are connected by AraP, which transports arabinobiose (and potentially other arabino-oligosaccharides) originating from arabinan catabolism into the cytoplasm (Fig. 2 and 5B) and is encoded in the arabinose utilization locus (Fig. 1).

We established that BT4338 is a global regulator required for full transcriptional activation of the genes necessary to metabolize both arabinan and arabinose (Fig. 6), utilization of several carbohydrates (see Fig. S7A to G in the supplemental material), and wild-type growth kinetics on other carbohydrates (see Fig. S7L to V). Our *in vivo* analysis provides direct genetic evidence for regulatory interactions suspected on the basis of biochemical (11) and bioinformatics (10) analyses.

Taken together, our findings establish that *B. thetaiotaomicron* coordinates the utilization of arabinan, arabinose, and other nutritional signals. These coordinated processes may play a critical role in gut colonization because *BT4338* and several genes within the arabinan PUL and arabinose utilization operon are necessary for survival in the murine gut (6, 12).

### Bacteria use distinct strategies for arabinan and arabinose utilization.

Arabinan utilization is restricted to a subset of microorganisms, including *Aspergillus spp.* (17); Gram-positive soil bacteria, such as *Bacillus subtilis* (18); and mammalian gut *Bacteroidete*s (4, 10). *B. subtilis* encodes several *endo*-arabinases and α-l-arabinofuranosidases that catabolize arabinan to arabinose (18, 19). The genes specifying these enzymes are regulated solely by a distinct AraR protein that senses intracellular arabinose (18).

By contrast, arabinan breakdown in the Gram-negative bacterium *B. thetaiotaomicron* is regulated by an activator (BT0366) that senses a degradation product of arabinan in the periplasm, a repressor (AraR) that senses arabinose in the cytoplasm, and a global regulator (BT4338) that senses a yet-undescribed signal. The strategy for arabinan breakdown in *B. thetaiotaomicron* is reminiscent of the enzymatic capabilities of *B. subtilis* arabinan utilization and the regulatory framework governing arabinose utilization in *E. coli*, which relies on AraC operating both as an activator and as a repressor (3).

### A new regulatory paradigm for polysaccharide utilization in *Bacteroides.*

We present a new regulatory paradigm governing utilization of arabinan and arabinose that relies on sensing both a degradation product of the polysaccharide and the monosaccharide (Fig. 1). We propose that variations on this paradigm are more common in the *Bacteroidetes* than the existing paradigm where a single regulator senses monomeric fructose and activates transcription of genes necessary for utilization of both levan and its constituent fructose (7). This is because of the following. (i) The regulator that senses fructose (BT1754) is thus far unique among the *Bacteroidetes* in sensing a monomeric sugar in the periplasm (7). Indeed, several characterized regulators sense polymers of two to eight monosaccharides in length (4, 20, 21). (ii) Several genes in *B. thetaiotaomicron* and other *Bacteroides* spp. are predicted to encode regulators that sense cytoplasmic monosaccharides (10, 11, 22, 23). (iii) The monosaccharides sensed by these regulators are components of complex dietary and mucosal polysaccharides encountered by *Bacteroides* in the gut (14). (iv) Bioinformatics analysis predicts that some of these regulators bind to promoters of genes not necessary for utilization of the monosaccharide that they sense (10).

### The pleiotropic transcriptional regulator BT4338.

BT4338 was previously designated MalR because a transposon insertion in the *BT4338* gene in a strain lacking a functional copy of the starch utilization regulator SusR decreased the ability of *B. thetaiotaomicron* to metabolize maltose and maltotriose (16). The domain structure of the BT4338 protein is similar to that of the *Proteobacteria* CRP (despite low sequence identity) with an N-terminal CRP/Fnr-like ligand-binding domain and a C-terminal DNA-binding domain (15, 24). CRP also controls expression of genes involved in adhesion (25, 26) and virulence (27, 28) in *Proteobacteria*. We have now determined that the pleiotropic regulatory protein BT4338 is necessary for utilization of multiple sugars (see Fig. S7 in the supplemental material). Therefore, BT4338 may connect expression of arabinan and arabinose utilization genes to a larger regulatory network that integrates polysaccharide utilization with additional metabolic signals and/or other physiological cues in *B. thetaiotaomicron*. The identification of the signal(s) controlling the levels and activity of the BT4338 protein may help us understand its critical role in the colonization of the mammalian gut (6).

## MATERIALS AND METHODS

### Bacterial strains and growth conditions.

*B. thetaiotaomicron* strains were derived from strain VPI-5482 (15) and grown under anaerobic conditions at 37°C on brain heart infusion agar supplemented with 10% horse blood and in tryptone-yeast extract-glucose medium containing tetracycline (2 µg/ml), erythromycin (10 µg/ml), gentamicin (200 µg/ml), or 5-fluro-2′-deoxyuridine (FUdR) (200 µg/ml), when needed. All experiments with *B. thetaiotaomicron* were performed with cells grown anaerobically in minimal medium (9) supplemented with the indicated carbon sources and antibiotics when required. *E. coli* strains were derived from S17-1 and grown in LB medium containing 100 µg/ml ampicillin. All chemicals were purchased from Sigma except arabinan (sugar beet, P-ARAB), arabinobiose (O-ABI), pectic galactan (P-PGAPT), and rhamnogalacturonan I (P-RHAM1), which were purchased from Megazyme, and beta-d-(−)-fructose (MP Biomedicals). Dialyzed arabinan was prepared by incubating 10 ml of 5% (wt/vol) arabinan within a 3,500-molecular-weight-cutoff (MWCO) Slide-A-Lyzer dialysis cassette (Thermo) in 1.5 liters distilled water overnight at 4°C. All strains and plasmids used in this study are listed in Table S1 in the supplemental material. All oligonucleotides used in this study are listed in Table S2 in the supplemental material.

### Strain construction.

Phusion high-fidelity polymerase was used to amplify all DNA fragments, which were ligated into vectors by T4 DNA ligase or NEBuilder HiFi DNA Assembly Master Mix (all products from NEB). Deletion mutants were generated using counterselectable allelic exchange (9).

### Growth curve analysis.

Growth of *B. thetaiotaomicron* strains was examined as follows. Following overnight incubation in tryptone-yeast extract-glucose (TYG) medium, bacteria were subcultured at a 1:500 dilution directly into the indicated medium. Growth proceeded anaerobically and was monitored by *A*_595_ measurement in a Tecan Infinite F200 Pro microplate reader. Growth rate was quantified by identifying the absorbance where growth increased by 15% over the baseline (*A*_min_) and the maximum growth immediately after exponential growth (*A*_max_). The time points corresponding to these absorbances, *T*_min_ and *T*_max_, respectively, were used to calculate growth rate as (*A*_max_ − *A*_min_)/(*T*_max_ − *T*_min_). To examine the effects of the *araR* mutation on growth adaptation to arabinan, cultures were grown overnight in minimal medium with 0.5% glucose and subcultured 1:50 into the same medium, and cells were grown to an optical density at 600 nm (OD_600_) of 0.3 to 0.4. Cells were harvested by centrifugation, resuspended in minimal medium lacking a carbon source, and incubated with 0.1% (wt/vol) arabinan or chondroitin sulfate.

### Gene expression analysis and quantitative real-time PCR.

Time course gene expression analysis was carried out as described previously (20), with the following modifications. Cells were grown to an OD_600_ of 0.35 to 0.5 prior to induction. Cells were harvested by centrifugation and resuspended in medium containing the indicated carbon sources. One-milliliter culture samples were collected before (−5-min time point) and at the indicated times after introduction to medium containing the indicated carbon sources. mRNA levels of genes were measured as described previously (29). mRNA levels are represented normalized to a 1,000-fold dilution of 16S rRNA abundance to account for cell density or as a fold change of values obtained from this normalization.

### Western blot analysis.

To examine BT0366 hemagglutinin (HA) protein levels, minimal medium cultures (10 ml) were harvested at each time point before (0.5% glucose) and after (0.1% arabinan) induction. Western blotting was performed as described previously (29). Membranes were immunoblotted with anti-HA (Sigma) or anti-*E. coli* DnaK (Clontech) antibodies.

### Electrophoretic mobility shift assays.

Electrophoretic mobility shift assays were carried out as described previously (30), with the following modifications. Fragments were amplified from *B. thetaiotaomicron* VPI 5482 genomic DNA. The BT0366 response regulator domain used for binding was purified as described previously (29).

## SUPPLEMENTAL MATERIAL

Figure S1  The *araP* gene contributes to growth in arabinose-containing polysaccharides. (A) Growth of the *BT0356*:pSAM (NS423) and wild-type (GT23) *B. thetaiotaomicron* strains in minimal medium containing 0.5% fructose. (B) Growth of the *araP* (NS401) and wild-type (GT23) *B. thetaiotaomicron* strains in minimal medium containing 0.5% arabinogalactan. Graphed are the mean and standard error of the mean from five independent replicates grown in the same plate. Download Figure S1, EPS file, 1.4 MB

Figure S2  (A) mRNA levels of the *araM* gene in the *BT0366* (GT44) and isogenic wild-type (GT23) strains prior to the switch (−5) and after 1- and 2-h exposure to minimal medium (MM) containing 0.1% 3.5-kDa dialyzed arabinan. Graphed are the mean and standard error of the mean from three independent experiments. (B) Growth of the *BT0366* (GT44) and isogenic wild-type (GT23) strains in minimal medium containing 0.5% arabinose. Graphed are the mean and standard error of the mean from six independent replicates grown in the same plate. Download Figure S2, EPS file, 1.4 MB

Figure S3  Nucleotide sequence of the promoter regions corresponding to the *BT0356*, *BT0365*, and *BT0366* to -*67* genes. Note that the *BT0366*-to-*BT0367* region is in the positive-sense orientation for *BT0367*. Boxed are the *in silico* predicted binding sites for AraR (red), BT0366 (green), and BT4338 (blue) (D. A. Ravcheev, A. Godzik. A. L. Osterman, and D. A. Rodionov, BMC Genomics **14:**873, 2013, http://dx.doi.org/10.1186/1471-2164-14-873). Boldface indicates the predicted −7 box sequences (E. H. Patel, L. V. Paul, S. Patrick, and V. R. Abratt, Res Microbiol **159:**678–684, 2008, http://dx.doi.org/10.1016/j.resmic.2008.09.002). Italics indicate predicted start codons. Brackets indicate the fragments used for electrophoretic mobility shift assays (Fig. 5A). Download Figure S3, EPS file, 0.4 MB

Figure S4  AraR controls expression of arabinose and arabinan utilization genes. (A) mRNA levels of the *araM* and *BT0364* genes in a *B. thetaiotaomicron* strain deleted for *araR* complemented with *araR* expressed from the *araM* promoter in single copy on the chromosome (araR + pNBU: *araR*, NS441) or the empty vector (araR + pNBU, NS440) and the isogenic wild-type strain with the empty vector (WT + pNBU, VR86) at steady state in minimal medium (MM) containing 0.5% glucose. Graphed are the mean and standard error of the mean from at least three independent experiments. (B) Growth of the *araR* (NS367) and isogenic wild-type (GT23) strains in MM containing 0.5% arabinan. Graphed are the mean and standard error of the mean from six independent replicates grown in the same plate. (C) mRNA levels of the *araM* and *BT0268* genes in the *araR* (NS367) and isogenic wild-type (GT23) strains prior to the switch (−5) and after 1- and 2-h exposure to MM containing 0.1% arabinogalactan. (D) mRNA levels of the *araM* and *BT4164* genes in the *araR* (NS367) and isogenic wild-type (GT23) strains prior to the switch (−5) and after 1- and 2-h exposure to MM containing 0.1% rhamnogalacturonan. (E) Growth of *araR* (NS367) or the isogenic wild-type (GT23) after switching from MM containing 0.5% glucose to MM containing 0.1% chondroitin sulfate. Graphed are the mean and standard error of the mean from at least three independent experiments. Asterisks indicate significant difference from the wild-type strain in panels C and D (*, *P* ≤ 0.05; **, *P* ≤ 0.01; ***, *P* ≤ 0.001 by two-tailed Student’s *t* test). Note log scale of *y* axis in panels A, C, and D. Download Figure S4, EPS file, 1.2 MB

Figure S5  (A) mRNA levels of the *BT0364* gene in isogenic *araP* (NS401) and wild-type *B. thetaiotaomicron* (WT, GT23) strains prior to the switch (−5) and after 1-h exposure to minimal medium (MM) containing 0.1% arabinose or 0.1% arabinan. Graphed are the mean and standard error of the mean from four independent experiments. (B) Growth of the *araR BT0366* (NS422) and isogenic wild-type (GT23) strains in MM containing 0.5% arabinan. Graphed are the mean and standard error of the mean from six independent replicates grown in the same plate. Note log scale of *y* axis in panel A. Download Figure S5, EPS file, 1.6 MB

Figure S6  The *BT4338* gene is necessary for utilization of arabinose and arabinan. (A) Growth of the *BT4338* (NS364) and isogenic wild-type (WT, GT23) strains in tryptone-yeast extract-glucose (TYG) medium. (B) Growth of the *BT4338* strain complemented with *BT4338* expressed from its native promoter in single copy on the chromosome (BT4338 + pNBU:*BT4338*, NS433) or the empty vector (BT4338 + pNBU, NS432) and the isogenic wild-type strain with the empty vector (WT + pNBU, VR86) in minimal medium (MM) containing 0.5% arabinose. (C) Growth of the strains listed in panel B in MM containing 0.5% arabinan. Graphed are the mean and standard error of the mean from six independent replicates grown in the same plate. Download Figure S6, EPS file, 2.1 MB

Figure S7  The *BT4338* gene is necessary for growth in a subset of carbohydrates. Growth of the *BT4338* (NS364) and isogenic wild-type (GT23) strains in minimal medium containing 0.5% arabinogalactan (A), fucose (B), glucuronate (C), *N*-acetylgalactosamine (D), polygalacturonic acid (E), ribose (F), xylose (G), glucose (H), heparin (I), mannose (J), *N*-acetylglucosamine (K), galactose (L), amylopectin (M), galacturonate (N), chondroitin sulfate (O), fructose (P), maltose (Q), maltotriose (R), α-mannan (S), pectic galactan (T), rhamnogalacturonan (U), or rhamnose (V). Graphed are the mean and standard error of the mean from five independent replicates grown in the same plate. Download Figure S7, EPS file, 2.7 MB

Table S1  Bacterial strains and plasmids used in this study. Table S1, DOC file, 0.1 MB

Table S2  Oligonucleotides used in this study. Table S2, DOC file, 0.1 MB
