# Supplementary material for: Multiple Signals Govern Utilization of a Polysaccharide in the Gut Bacterium Bacteroides thetaiotaomicron
Source: mBio. 2016 Oct 11;7(5):e01342-16. doi: 10.1128/mBio.01342-16 (PMC5061871; doi:10.1128/mBio.01342-16)
Supplement: Table S1 — Bacterial strains and plasmids used in this study. [file mbo005163020st1.doc]

Table S1. Bacterial strains and plasmids used in this study.

| Plasmids | | |
| --- | --- | --- |
| Plasmid | Description | Reference |
| pNBUtet | *Bacteroides* chromosomal vector | (1) |
| pExchange-*tdk* | *Bacteroides* deletion vector | (1) |
| pGT12 | pT7-7: BT0366 RR | (2) |
| pGT26 | pExchange-*tdk*: Flanks up and downstream of *BT0366* surrounding a XmaI site | This work |
| pNS201 | pNBUtet: P(*BT0366*): *BT0366*HA | This work |
| pNS272 | pExchange-*tdk*: Flanks up and downstream of *BT0354* | This work |
| pNS359 | pExchange-*tdk*: Flanks up and downstream of *BT4338* | This work |
| pNS400 | pExchange-*tdk*: Flanks up and downstream of *BT0355* | This work |
| pNS425 | pNBU2-tet::P(*malR*): *BT4338*HA | This work |
| pNS431 | pNBU2-tet::P(*araM*): *BT0354* | This work |
| Bacterial Strains | | |
| *Escherichia coli* | | |
| Strain | Description | Reference |
| S17-1 | IncP RP4 inserted into the chromosome | (3) |
| *Bacteroides thetaiotaomicron* | | |
| Strain | Description | Reference |
| VPI-5482 *tdk* | Δ*tdk* | (1) |
| GT44 | Δ*tdk* Δ*BT0366* | This work |
| VR86 | Δ*tdk* pNBUtet | (4) |
| NS204 | Δ*tdk* Δ*BT0366* pNBUtet: P(*BT0366*): *BT0366*HA | This work |
| NS364 | Δ*tdk* Δ*BT4338* | This work |
| NS367 | Δ*tdk* Δ*BT0354* | This work |
| NS401 | Δ*tdk* Δ*BT0355* | This work |
| NS404 | Δ*tdk* Δ*BT0354* Δ*BT4338* | This work |
| NS408 | Δ*tdk* Δ*BT0354* Δ*BT0366* Δ*BT4338* | This work |
| NS422 | Δ*tdk* Δ*BT0354* Δ*BT0366* | This work |
| NS423 | *BT0356*:pSAM | (5) |
| NS432 | Δ*tdk* Δ*BT4338* pNBUtet | This work |
| NS433 | Δ*tdk* Δ*BT4338* pNBUtet: P(*malR*): *BT4338*HA | This work |
| NS440 | Δ*tdk* Δ*BT0354* pNBUtet | This work |
| NS441 | Δ*tdk* Δ*BT0354* pNBUtet: P(*BT0356*): *BT0354* | This work |

1. **Koropatkin NM, Martens EC, Gordon JI, Smith TJ.** 2008. Starch catabolism by a prominent human gut symbiont is directed by the recognition of amylose helices. Structure **16:**1105-1115.

2. **Townsend GE, 2nd, Raghavan V, Zwir I, Groisman EA.** 2013. Intramolecular arrangement of sensor and regulator overcomes relaxed specificity in hybrid two-component systems. Proc Natl Acad Sci U S A **110:**E161-169.

3. **Cho KH, Salyers AA.** 2001. Biochemical analysis of interactions between outer membrane proteins that contribute to starch utilization by *Bacteroides thetaiotaomicron*. J Bacteriol **183:**7224-7230.

4. **Raghavan V, Lowe EC, Townsend GE, 2nd, Bolam DN, Groisman EA.** 2014. Tuning transcription of nutrient utilization genes to catabolic rate promotes growth in a gut bacterium. Mol Microbiol **93:**1010-1025.

5. **Goodman AL, McNulty NP, Zhao Y, Leip D, Mitra RD, Lozupone CA, Knight R, Gordon JI.** 2009. Identifying genetic determinants needed to establish a human gut symbiont in its habitat. Cell Host Microbe **6:**279-289.
