# Supplementary material for: Multiple Signals Govern Utilization of a Polysaccharide in the Gut Bacterium Bacteroides thetaiotaomicron
Source: mBio. 2016 Oct 11;7(5):e01342-16. doi: 10.1128/mBio.01342-16 (PMC5061871; doi:10.1128/mBio.01342-16)
Supplement: Table S2 — Oligonucleotides used in this study. [file mbo005163020st2.doc]

Table S2. Oligonucleotides used in this study.

| Primer Number | Primer Name | Oligonucleotide Sequence |
| --- | --- | --- |
| Constructs for *Bacteroides* mutants | | |
| 10252 | pEX-0366 5'F BamHI | CAGGATCCCGATAGCATTCATCGCGGTA |
| 10342 | pEX-0366 5R XmaI | GCCCCGGGATATGTACGTATTAATAAGG |
| 10343 | pEX-0366 3F XmaI | GCCCCGGGCTGATATAAGGGTTGCTATT |
| 10344 | pEX-0366 3R SalI | GCCGTCGACATTGCGTTCGTTACACTGAC |
| 10433 | Pbt0366 fwd BamHI | CGGATCCCAATTATGCTACAAAAATAC |
| 13822 | R0366HA-2 | GATTGTCGACTTAAGCGTAGTCTGGGACGTCGTATGGGTATTCTTCGTTATTCAGCAGTG |
| W1225 | d0354-1 | CATggattcAAGCTGTTGCCTGTATTCCTG |
| W1226 | d0354-2 | CAGCATATTGACGTTTGAATGAATTTATTTAATTAC |
| W1227 | d0354-3 | TTCATTCAAACGTCAATATGCTGGAAGAACTGAAA |
| W1228 | d0354-4 | CATGTCGACTATAAATTCACAATCCGAACAGC |
| W2642 | d4338-1 | GCTCTAGAACTAGTggatccTCAGTGGAGAAACTGATGGA |
| W2643 | d4338-2 | GGGTGCTACAAAACTGTGTTATAC |
| W2644 | d4338-3 | aacacagttttgtagcacccTACAGACCTTATACGATCATAAAAAGT |
| W2645 | d4338-4 | AAGATAACATTCGAgtcgacAGCATAAGCTACCACCTCTTT |
| W2856 | d0355-1 | GCTCTAGAACTAGTGGATCCCAGCATTGCTGATTATATTAATGTA |
| W2857 | d0355-2 | AATAAATTTGATTTATAAGTTAATATTATTTCTCT |
| W2858 | d0355-3 | ACTTATAAATCAAATTTATTTTAAATAAATTCATTCAAACGATG |
| W2859 | d0355-4 | AAGATAACATTCGAGTCGACTACCATATCTTCCGCTTTCATAT |

| Primer Number | Primer Name | Oligonucleotide Sequence |
| --- | --- | --- |
| W2873 | BT4338haR | AAGATAGGCAATTAgtcgacTTAAGCGTAGTCTGGGACGTCGTATGGGTAtcctatcttgcttattttcttgag |
| W2935 | PmalRF | GCTCTAGAACTAGTggatccTCAAAGTACTGGTACGCGAAATGA |
| W2942 | P0356F | GCTCTAGAACTAGTggatccGTCCATTCGTTTCCATATGC |
| W2946 | pN0354R | AAGATAGGCAATTAgtcgacTTATAATTTAAATTTCGGGTCCTT |
| Real-time PCR | | |
| 10203 | BT_0367 RT FWD | GAACCGGAAAGAAGCGAGAGA |
| 10204 | BT_0367 RT REV | AACGGCGCATCTGGATAAAT |
| 10256 | 16s qRT-PCR fwd | GGTAGTCCACACAGTAAACGATGAA |
| 10257 | 16s qRT-PCR rev | CCCGTCAAATTCCTTTGAGTTTC |
| 10667 | BT_0268-rtF | GCCCGGTGTATATGTACAGGAAA |
| 10668 | BT_0268-rtR | CACGAACCAGCATGCTTTGA |
| 13331 | qBT0366HKf | TGTCACGCCAAAACCAGATG |
| 13332 | qBT0366HKr | TGCCTTCTGCCTCGTGATTT |
| 13559 | qBT0364F | CAGGACGAATACGGAAACAAGTC |
| 13560 | qBT0364R | GTCGAGATTGGCACGGAAAG |
| W1784 | qBT0356F | AACGGCAACGGATATGATCAC |
| W1785 | qBT0356R | GCTACCTGCGAGATGTCACCTT |
| W3214 | qBT4164F | CTGCCAAATACATCGGTCTGAA |
| W3215 | qBT4164R | TTGCAGGAGCATCAGACAACA |
| Electrophoretic Mobility Shift Assay (EMSA) Primers | | |
| 10056 | BT_0366 PROMOTER Fwd | AGATACTATATATTATAAGG |
| 10057 | BT_0366 PROMOTER Rev | TGCTACTAAAAGAACTGGCA |
| 10058 | BT_0365 PROMOTER Fwd | GGGTTGCTATTATGAAACAAT |
| 10059 | BT_0365 PROMOTER Rev | GAACGGTAAGAATTGCCATA |
| W2937 | dBT0356-2 | GGTTTTATTTTTAATGTAAATGAGATG |
| W2941 | F0356EMSA | AACTATCCAGCTCTATATAGAAAGAGA |
